# Manuscript reviews—an evolving challenge

**DOI:** 10.1093/jhps/hnaf068

**Published:** 2025-12-18

**Authors:** Richard E Field

**Affiliations:** City St George’s University of London, London, United Kingdom

On Monday 5 January 1665, the French lawyer, Denis de Sallo, became the world’s first journal editor with the publication of the first issue of the weekly *Journal des sçavans* [1]. The journal focused on philosophy, theology, law, and science. Denis de Sallo’s editorial tenure only lasted three months as some of the journal content incurred King Louis XIV’s displeasure. After a short hiatus publication resumed under Jean Gallois, a scholar and cleric, who ensured that the journal’s tone was more acceptable to the authorities. Inspired by the French initiative, Henry Oldenburg [2], a founding member the Royal Society, became the first Editor of the Philosophical Transactions of the Royal Society, with the first issue published in March 1665*.* One of Oldenburg’s initiatives was to introduce an early form of peer review, sending submissions to trusted experts for advice before printing.

During the nineteenth and twentieth centuries manuscript evaluation evolved to provide the double-blinded, peer-review model that is the mainstay of many contemporary medical and scientific journals. For all its merits, the peer-review process also has shortcomings. The review process is often frustratingly slow. The editorial team must identify suitably qualified reviewers and persuade them to review the manuscripts. Neither step is straight-forward and journal editors often bemoan the number of reviewer invitations they need to make to secure two seemingly acceptable reviews. However, there is no guarantee that reviews have been undertaken with due diligence, provide a sufficiently expert opinion, are impartial or valid. In a number of unfortunate cases, advice from reviewers has persuaded editors to reject manuscripts detailing work that subsequently secured a Nobel prize [3].

Over the last few decades, innovations such as Open Peer Review [4, 5], Post-Publication Peer Review [6, 7], Preprint [8], and Community Review Ecosystems have gained some traction. It remains to be seen whether AI technology will assist or supersede the adoption of these models. Publisher guidance on the role of AI is also evolving from initial wariness to a growing recognition that the adoption of AI technology is inevitable. In this rapidly shifting environment, the current position of most publishers is to remind authors and researchers that it is the authors who are responsible and accountable for the contents of their work.

The shift in attitude to AI technology is clearly seen in the development and adoption of AI tools that can accelerate evaluation and publication of submissions. Such tools can assist in the initial screening and triage process to analyse submitted manuscripts for completeness, formatting compliance, and to assess whether the manuscript matches the journal’s scope [9–11]. Machine learning tools have been developed to compare how keywords, author details, and citation analysis compare with already accepted papers [12]. Elsevier have adopted an AI tool [13] that analyses their Scopus database [14] to help editors identify appropriate peer reviewers by analysing publication records, subject keywords, reference lists, and prior review history. AI tools have also been adopted to undertake integrity checks as well as plagiarism and duplication detection [11, 15]. Though less discussed in scholarly publishing than in journalism, AI tools can also be used for language polishing, formatting checks, and to ensure that references are consistent [16].

The adoption of AI technology also carries risk. Human judgement remains critical to editorial decision-making [11, 16, 17]. If a manuscript is uploaded by an author, reviewer, or editor for analysis using an AI tool the act of uploading the manuscript may grant to the AI tool rights to the input materials and potentially also constraints on the use of the AI tool’s outputs that could restrict the subsequent publication of the material. AI models may reproduce or amplify biases (e.g. geographic, language, gender). AI may struggle with deeper conceptual evaluation: novelty, significance, methodological nuance [16]. Authors, reviewers, and editors should be mindful that failure to disclose that an AI tool was used and how it was used may violate a journal’s policy.

The role of AI is evolving. While many publishers initially exhibited considerable wariness over the use of AI tools by researchers and authors, it is inevitable that their rules will change and journals will adapt as AI tools enhance our ability to generate and validate research manuscripts.

The potential value of AI tools to shorten the manuscript submission to publication time is well exemplified by the papers included in this issue. Excluding the paper from Chen and his colleagues [18], which ran into unusual and protracted administrative delays, the average duration from submission to acceptance was 221 days (range 89–405 days). The average interval between acceptance and e-publication was considerably shorter at 35 days (range 6–77 days). Six of the papers included in this issue were accepted after a single revision, one required two revisions [19], and one required three revisions [20]. In the last case, the requirement for three revisions was further complicated by the need to invite 10 reviewers before two useful reviews were returned. These challenges almost doubled the interval from first submission to acceptance when compared to papers accepted after a single revision. Clearly, the greatest opportunity for reducing the submission to publication interval lies in shortening the manuscript review process; whether it be faster engagement of reviewers, securing reviews more quickly, or swifter action by the editorial team. These are the steps that would gain most benefit from AI tools. The case is strong for adoption of such tools so long as they can be shown to be effective and reliable.

In the last 3 years, over 300 people have kindly completed reviews of manuscripts submitted to the *Journal of Hip Preservation Surgery* (*JHPS*) and over a third of these people have reviewed three or more manuscripts. This represents a massive body of work and time commitment. The work has all been undertaken without personal reward beyond the knowledge that manuscripts have been improved and that authors and readers have benefitted from our reviewers’ considered and valuable feedback. We are enormously grateful to everyone who has helped make *JHPS* the premier journal for the hip preservation community and we will do our best to ensure that work submitted to the journal is processed as efficiently and fairly as possible.
